# Safety and effectiveness of primary transscleral diode laser cyclophotoablation for glaucoma in Nigeria

**DOI:** 10.1111/ceo.13328

**Published:** 2018-06-17

**Authors:** Mohammed M Abdull, David C Broadway, Jennifer Evans, Fatima Kyari, Fatima Muazu, Clare Gilbert

**Affiliations:** ^1^ Ophthalmology Department Abubakar Tafawa Balewa University Teaching Hospital Bauchi Nigeria; ^2^ Department of Clinical Research London School of Hygiene and Tropical Medicine London UK; ^3^ Directorate of Ophthalmology Norwich and Norfolk University Hospital NHS Foundation Trust Norwich UK; ^4^ Baze University Abuja Nigeria

**Keywords:** Africa, cyclophotocoagulation, diode laser, glaucoma

## Abstract

**Importance:**

To investigate the safety, effectiveness and follow‐up rates after transscleral diode laser cyclophotocoagulation as primary treatment for seeing eyes with primary open angle glaucoma in Bauchi, Nigeria.

**Background:**

There is a high prevalence of primary open angle glaucoma in Africa where adherence to medical treatment and acceptance of surgery are poor.

**Design:**

Prospective case series.

**Participants:**

New glaucoma patients where surgical intervention was recommended.

**Methods:**

A diode 810 nm laser G‐probe was used under retrobulbar anaesthesia to deliver approximately 20 shots for 2000 ms, titrating the power. If both eyes were treated the first was the study eye. Repeat treatment offered if the intraocular pressure (IOP) was >21 mmHg on two consecutive visits.

**Main Outcome Measures:**

IOP < 22 mmHg, change in ≥2 lines of Snellen visual acuity (VA), and complications.

**Results:**

201 out of 204 eyes with complete data analysed. Mean age 52 years, 17 (8.3%) eyes were re‐treated. Mean pre‐treatment IOP was 39 (SD 11) mmHg. 106 (53%) attended at 12 months when the mean IOP was 19 (7–45) mmHg; 77 (73%) had IOP < 22 mmHg. VAs were better in 13 (12.3%) and worse in 23 (21.7%) eyes. Postoperative complications included mild uveitis (5.5%), corneal oedema (2.5%), severe uveitis (0.5%) and transient hypotony (2.0%). No hypotony at 12 months.

**Conclusions and Relevance:**

Transscleral diode laser cyclophotocoagulation controlled IOP in almost three quarters of eyes at 12 months with short‐term preservation of vision and minimal complications. Poor follow‐up in this setting highlights the need for an effective, safe and acceptable treatment where regular follow‐up is less critical.

## Introduction

Africa is the region with the highest prevalence of primary open angle glaucoma (POAG) affecting an estimated 7 million people aged 40–80 years.^1^ The predisposition to POAG in Africa is likely due to variation in genetic susceptibility.^2^ Glaucoma is responsible for a higher proportion of blindness in Africa than in other regions (range 8–22.9%)^3^, ^4^, ^5^ being 16.3% in Nigerian adults aged 40 years and above.^6^

There are two disease‐related factors which increase the life‐time risk of blindness from glaucoma in Africa: an earlier age of onset^7^, ^8^ and a more aggressive course.^9^ In Africa, most people with glaucoma present very late, often blind in one eye^10^, ^11^ and there is poor awareness of the disease.^12^, ^13^ Services for eye care, particularly for primary eye care and specialist glaucoma care, are inadequate and mainly located in cities.^14^, ^15^ A high proportion of the population are poor and cannot afford the cost of treatment or follow‐up,^16^ and adherence to systemic or topical medication is often low.^11^, ^17^ Acceptance of glaucoma surgery is also low, <2% in a study in the same hospital as this case series,^11^ as it does not improve visual function, and patients fear surgery on their only seeing eye. Although trabeculectomy can provide stable, long‐term intraocular pressure (IOP) control in people of African origin,^18^ ophthalmologists in Africa^19^ are often reluctant to offer trabeculectomy for fear of complications, including visual field ‘wipe‐out’ in advanced cases^20^ and the variable outcomes.^21^, ^22^, ^23^, ^24^, ^25^

There are only a few studies comparing outcomes of surgical interventions for glaucoma in patients of African descent, including laser procedures.^26^, ^27^ In a recent review, the authors concluded that there was no evidence that any procedures are superior to trabeculectomy, and there is compelling evidence that the outcomes of trabeculectomy are less good than for Caucasian eyes, particularly if antimetabolites are not used.^28^ Given the relatively low uptake of surgery, poor outcomes and inadequate follow‐up, laser treatment could be considered as a primary treatment for glaucoma in Africa despite the limited reported evidence.

In a review of 18 studies of transscleral diode laser cyclophotocoagulation as (TDLC) treatment, the number of eyes treated ranged from 8 to 263, and follow‐up was 9–66 months.^29^ The studies had different indications for treatment and often included different types of glaucoma. The proportion of eyes in which intraocular (IOP) was controlled (i.e. <22 mmHg) ranged from 38% to 88.1%.^29^ There are only a few studies of TDLC for seeing eyes, or as a primary treatment, or which were undertaken in Africa.

With respect to studies on seeing eyes (see Tables S1 and S2, Supporting information), in a retrospective study in the United Kingdom, the indication for treatment was uncontrolled glaucoma. Forty‐six eyes were treated, 52% had POAG and the mean pre‐treatment IOP was 24 (12–35 mmHg). At 2 years, 80% of eyes had an IOP of <22 mmHg with or without additional topical treatment: 23.9% of eyes lost more than two lines of VA.^30^ In another UK study, 49 seeing eyes were treated for uncontrolled glaucoma and at 5 years IOP was controlled (6–21 mmHg) in 79.6% of eyes; 30.6% lost ≥ 2 lines of VA.^31^

Primary TDLC treatment has been reported in several studies (Tables S1 and S2). For example, a study in Germany recruited individuals who refused surgery or where follow‐up could not be guaranteed: among the 25 eyes treated, re‐treatment was required in three eyes.^32^ In another study for a range of different types of glaucoma in Germany, 193 eyes were treated: at follow‐up 90% of eyes with POAG had IOPs of 10 to 22 mmHg after single or multiple treatments.^33^

There are only four studies reporting TDLC treatment outcomes in Africa, from Cameroon,^34^ Malawi,^35^ Ghana^36^ and Tanzania,^37^ which had different indications for treatment, varying outcomes, small sample sizes and poor follow‐up.

The studies demonstrate that TDLC laser treatment is relatively safe, with mild postoperative uveitis being the commonest complication (see Table S1). Other less common complications, such as conjunctival or scleral burns, hyphaema, atonic pupil, choroidal detachment, hypotony and visual loss, were more common in studies of patients with intractable or complex glaucoma.

Given the encouraging results of TDLC in seeing eyes and the need for an acceptable one‐off treatment in Africa, a prospective study of TDLC was undertaken for POAG in seeing eyes as an alternative to standard care. The purpose of the study was to explore the safety and effectiveness of TDLC in terms of IOP lowering, and to provide data on the rate of follow‐up at 1 year. All the findings will be used to design a clinical trial. The study was undertaken in a university teaching hospital in north‐east Nigeria.

## Methods

All patients provided written informed consent for the procedure. The study adhered to the tenets of the Declaration of Helsinki.

Glaucoma was diagnosed on the basis of vertical cup–disc‐ratio (VCDR), IOP and visual field analysis, where possible. Presenting visual acuity (VA) was measured in each eye using a Snellen E chart and categorized using World Health Organization definitions. Consecutive new patients with a range of severity of POAG but who had a VA of three out of 60 or better in one or both eyes and where surgical treatment was the treatment of choice, were recruited for primary TDLC. The following patients were excluded: previous glaucoma surgery, mature cataract, diabetic retinopathy, corneal opacities, those already bilaterally blind from glaucoma (VA < 3/60), and those who preferred topical medication. Patients treated with TDLC for blind, painful eyes were also excluded. Given the lack of nomenclature for lasers in the local language the procedure was described in Hausa as ‘special computer light treatment’. Data are presented on individuals who had a presenting VA of 3/60 or better in the treated eye and who were followed up for 12 months.

TDLC treatment was performed under retrobulbar anaesthesia with lignocaine and adrenaline 2% in the operating theatre using the G‐probe of the Iridex diode 810 nm laser (IridexCorporation.1212, Terra Bella Avenue Mountain View, CA, USA) in continuous mode. The probe heel was placed at the edge of the limbus matching the contour of the scleral curvature so that the small 0.7 mm protrusion indented the sclera approximately 1.2 mm posteriorly to optimize energy delivery to the ciliary body. Transillumination was not routinely used. Approximately 20 shots were delivered for 2000 ms. The power was reduced by 50 mW from the last audible pop to reduce the risk of inflammation and postoperative hyphaema.^38^ Treatment was given over 360°, avoiding the ciliary vessels at 3 and 9 o'clock, sub‐conjunctival dexamethasone 2 mg was given and the eye padded for 4 h. Oral diclofenac potassium 50 mg was prescribed twice a day and G dexamethasone 0.1% four times a day for 1 month, tailing off thereafter over a few weeks. On the first postoperative day, VA was measured using a Snellen E chart, patients underwent slit‐lamp biomicroscopy and IOPs were measured using Goldmann applanation tonometry.

Patients were reviewed at 1 day, 1 week and at 1, 4, 6 and 12 months when VA and IOPs were measured and anterior segments were examined at the slit lamp for complications. Patients were given dates for follow‐up but were not actively traced. If the IOP was raised (>21 mmHg) topical medication was initiated and if the IOP was still high at the next visit TDLC re‐treatment was offered. A second session of laser was given to those who consented.

The outcomes of the study were IOP control, defined as less than 22 mmHg and > 30% IOP reduction from presenting values, measured on the day laser treatment was offered.^29^, ^39^ Other outcomes were change in VA, defined as at least two lines change in Snellen VA, and complication rates. Uveitis was defined as mild if there was anterior chamber flare, or severe if flare and cells were present.

If both eyes were treated, the first eye was the study eye. Data were entered into a database created in Epidata and exported into Stata/IC 14.1 statistical software (StataCorpLP, College Station, TX, USA) for analysis. Follow‐up, IOP and VA findings at presentation and on the first postoperative day, at 1 week and at 1, 4, 6 and 12 months are presented. We calculated a Pearson's correlation coefficient to assess the relationship between change in IOP and presenting IOP.

## Results

A total of 204 seeing eyes (of 204 patients) with glaucoma that had not previously had surgical or laser treatment underwent TDLC. About 201 eyes were included in the analysis as presenting IOP data were missing for three. Seventeen (8.3%) eyes were re‐treated. The average power setting was 1770 mW (range 1100–2300 mW) with duration of 2000 ms. The average number of laser spots was 20 (range 15–25) per eye.

The mean age of the 201 patients was 52 (range 12–85) years and 69% were male. The mean VCDR in treated eyes at presentation was 0.9 with 44% having a VCDR of 1.0. Visual field analysis was only possible in 65 (32%) eyes. Not all patients attended every follow‐up appointment. A total of 106 (53%) attended at 12 months (Table 1). There were no differences in the age, sex or mean presenting IOP between those who attended the 1 year follow‐up and those who did not (Table 1).

**Table 1:** Comparison between patients followed up and not followed up at 1 year

	Followed up *n* = 106	Not followed up *n* = 95
Mean age in years (range)	51 (12–83)	52 (14–85)
Male *n* (%)	72 (68%)	66 (69%)
Mean presenting IOP, mmHg (range)	38 (22–72)	39 (22–70)

IOP, intraocular pressure.

### Intraocular pressure

The mean IOP before treatment (201 eyes) was 39 (SD 11; range 22–72) mmHg (Table 2) and the median was 37 (interquartile range 29–46) mmHg. On the first postoperative day, the mean IOP was 12 mmHg. IOPs at week 1, and after 1, 4 and 6 months were 11, 15, 18 and 19 mmHg, respectively. At 12 months, mean IOP among the 106 (53%) patients who attended was 19 (range 7–45) mmHg: 72.6% (77 out of 106) of eyes had an IOP of <22 mmHg and 83% had a drop in IOP of >30%. The proportion of eyes on topical glaucoma medication at follow‐up ranged from 1–11% over the 12 months, being 9% at 12 months. Follow up of the 17 re‐treated eyes was 41% (seven eyes) at 12 months. In the re‐treated eyes, mean IOP was 22 mmHg, 57% had an IOP of <22 mmHg and two were on topical medication.

**Table 2:** Presenting and postoperative IOP and topical medication use after TDLC treatment

Time period	Eyes	Follow up	IOP			IOP < 22 mmHg		On topical medication		IOP drop > 30%	
	*N*	%	Mean	SD	Range	*N*	%	*N*	%	*N* (%)	Mean (range) %
Presenting	201	100	39	11	22–72	201	100	0	0	0	0
1 day	191	95	12	5	2–30	186	97.4	1	1	188 (98)	−67 (−37/−94)
1 week	177	88	11	5	1–28	170	96.1	5	3	176 (99)	−68 (31–97)
1 month	156	78	15	7	1–48	135	86.5	15	10	144 (92)	−59 (−14–97)
4 month	131	65	18	8	2–50	108	82.4	15	11	117 (89)	−52 (−11–97)
6 month	118	59	19	8	3–52	89	75.4	13	11	103 (87)	−51 (−23–92)
12 month	106	53	19	7	7–45	77	72.6	10	9	88 (83)	−48 (−7‐87)

IOP, intraocular pressure.

**Figure 1:**
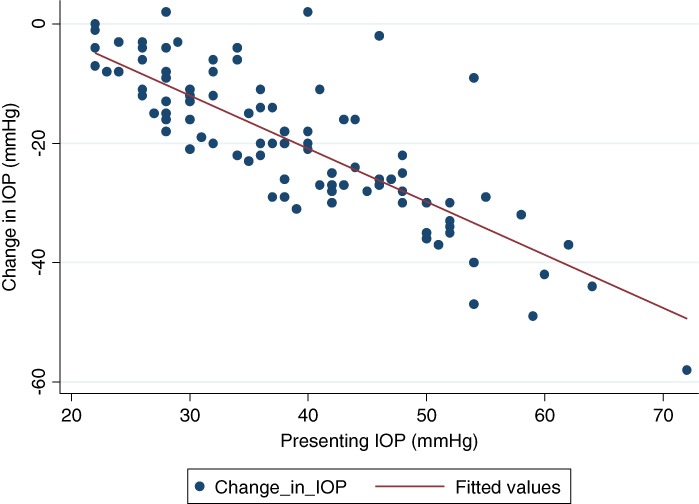
Scatterplot of change in IOP between presentation and follow up at 12 months, and presenting IOP in 106 eyes.

There was a strong correlation between change in IOP between presentation and follow up at 12 months, and presenting IOP (correlation coefficient 0.44, *P* < 0.001)(Fig. 1).

There was no significant associations between presenting and final IOP with the laser energy used nor the number of laser spots delivered. There were no age or gender differences in IOP at presentation or at 12 months.

### Visual acuity

The majority of eyes (83 out of 106, 78%) either retained their presenting VA (70, 66%) or the VA had improved by two or more lines at 12 months (13, 12%). VA deteriorated by two or more lines in 23 eyes (22%): these eyes had slightly higher pre‐treatment IOPs (mean 41; range 26–72 mmHg) than those not losing VA (mean 37; range 22–60 mmHg). At 12 months the mean IOP in the eyes that lost VA was 29 (range 8–45) mmHg compared with 19 (range 7–30) mmHg in eyes with stable VA. In addition, 12 (52%) eyes losing VA had a pre‐treatment VCDR of 1.0 compared with 28 (39%) of eyes not losing VA. Four eyes losing VA had progression of cataract, and six had corneal oedema before TDLC which persisted after treatment. There were no new cases of corneal oedema. Visual loss in the remaining eyes was attributable to glaucoma progression.

### Safety and complications

A few patients had mild anterior uveitis on the first postoperative day, which resolved with topical steroids (Table 3). No eyes developed hyphaema or other serious complications. Transient hypotony (IOP <6 mmHg), developed in four eyes during follow‐up but no eyes had hypotony at 12 months. Eyes that were re‐treated had slightly higher complication rates, with one eye developing severe uveitis.

**Table 3:** Complications after first and second treatment with transscleral diode laser cyclophotocoagulation

Complications	First treatment		Second treatment	
	(201 eyes)		(17 eyes)	
	*N*	%	*N*	%
Mild anterior uveitis	11	5.5	2	11.8
Severe uveitis	1	0.5	1	5.9
Hypotony (<6 mmHg) – temporary	4	2.0	0	0
Hypotony (<6 mmHg) – persistent	0	0.0	0	0
Corneal oedema	5	2.5	1	5.9
Cataract progression	4	2.0	0	0

## Discussion

This is the largest case series of TDLC as a primary treatment for seeing glaucomatous eyes in Africa. Unlike other studies from Africa the inclusion criteria were clearly defined, as were the outcomes. A standard treatment protocol was used and one ophthalmologist who had been trained in TDLC in the United Kingdom treated all the patients. TDLC was effective at attaining IOP < 22 mmHg in a high proportion of treated eyes with follow‐up data at 12 months, the majority not requiring additional treatment, with good preservation of VA in the short term. Our study suggests that the proportion of eyes with an IOP <22 mmHg is a better outcome measure in this population, where values at presentation were high, than the proportion achieving >30% reduction in IOP.

Our findings need to be considered in the context of the glaucoma patients who present to ABTUTH most of whom have advanced glaucoma, that is, they rarely accept incisional surgery nor adhere to topical medication or regular follow‐up after surgery. Laser treatment was, therefore, offered to patients where it was considered the only viable alternative. As in other TDLC studies, immediate post‐treatment complications were minimal, but were slightly higher in re‐treated eyes.

There was good acceptance of TDLC, which may be explained by the term used to describe it, which avoided the local Hausa term for surgery, ‘fidar ido’, which means ‘butchering’. TDLC was described as a once‐off, but repeatable treatment, which is desirable in settings where there is a fear of surgery and hospitalization, and a culture of not attending follow‐up. For service providers TDLC is easy to learn and the solid‐state laser used is cheaper, and more reliable and versatile than other lasers.

Poor follow‐up is a challenge in glaucoma care in Africa, as in this study, where patients either did not return for follow‐up at all, or did so at irregular intervals. Despite this, in our study over half returned at 12 months which was a marked improvement from an earlier study in the same hospital.^11^ Re‐treated eyes had poorer follow up and poorer IOP control than eyes that were not re‐treated. However, poor follow‐up may have biased the findings, since those who did not return may have lost vision and hence faith in the service. However, patients in whom vision had stabilized or improved may have failed to return, believing that they were ‘cured’. Poor follow‐up emphasizes the need in rural Africa for a procedure that maintains IOP control, which has few postoperative complications and where regular follow‐up is less critical.

Comparison of our findings with other studies from Africa is difficult given the variation in study designs, indications for and methods of treatment, outcome definitions and follow‐up rates. For example, the study in Ghana was a clinical trial to assess different laser power settings. Treatment was offered as a primary treatment and 92 eyes were treated: at 3 months 38 of the 79 patients who attended (48%) had an IOP of 22 mmHg or less; 16 of these eyes had been re‐treated and topical medication was being used in 68 eyes.^36^ The study in Tanzania was a retrospective review of 179 treated eyes only 49 of whom had at least one follow‐up visit. At the 3–6 month follow‐up four out of 12 eyes had an IOP of ≤21 mmHg and nine eyes were re‐treated.^37^ The study in Cameroon used a 910 nm laser (not the usual 810 nm laser) to treat 272 eyes but only 26 (<10%) attended at 12 months when the average IOP reduction was 7.5 mmHg, that is, lower than in our study.^34^ In the Malawi study, a low dose of 900 mW was used to treat POAG and pseudoexfoliative glaucoma. At 3 months mean IOP had fallen from 38.5 to 35.6 mmHg. In 50% of treated eyes, the IOP returned to pre‐treatment levels.^35^ These poor outcomes are probably explained by the low power setting used. In the present study IOP control was defined an IOP of <22 mmHg, a target used in other studies. However, the natural history and optimal target IOP to control glaucoma in Africa is not known.

Most glaucoma patients of African ancestry lose vision rapidly if they are not treated or have poor IOP control.^40^ In our study the majority of treated eyes either maintained their presenting VA or their acuity improved. The latter has been reported before.^41^ The majority of patients lost vision from progression of age related conditions such as cataract, or progression of glaucoma in end‐stage eyes, rather than as a direct result of the procedure.

Complications following treatment were minimal in the present study, and compared well with other studies on seeing eyes, but severe uveitis was higher following re‐treatment, but numbers are small. TDLC has had a relatively bad press in industrialized countries, probably because it is usually offered as the treatment of last resort. Failure rates and high complications rates are much more likely in these eyes.^42^

A limitation of this study was that visual fields, VCDRs and other parameters were not used to monitor disease progression. There are several reasons for this. Firstly, reliable assessment of visual fields is very difficult among uneducated African patients, as many have extensive visual field loss and are not familiar with interacting with technology. Second, many patients had corneal oedema at presentation, which prevented optic disc imaging at presentation, and lastly, optical coherence tomography was not available, which would have provided objective data to monitor structural changes at the optic nerve head. However, monitoring optic disc change in eyes with very advanced disease would be challenging, as in the present study, where almost half the eyes had a cup:disc ratio of 1.0 before treatment.

The findings of the present study may be generalisable to other parts of Africa where challenges faced by most glaucoma patients are similar. TDLC appeared to be acceptable, provided reasonable IOP control after one or two sessions and preserved vision at least in the short term for patients who would otherwise be without treatment.

### Implications for service delivery/research

TDLC is a simple, quick and minimally invasive and could be delivered by general ophthalmologists as a primary treatment^43^ or as an alternative to surgery in low‐income settings.^44^ The low cost, acceptability and ease of delivering TDLC, offers some promise in the otherwise bleak landscape of glaucoma care in Africa. Given that a once‐off treatment is the desired approach to glaucoma control in Africa, and that acceptance of trabeculectomy (standard of care)^11^, ^19^ is very low in our setting, clinical trials are needed to compare the effectiveness, acceptability, cost and safety of other forms of laser treatment as a primary treatment for glaucoma. These trials need to use standard definitions of control in terms of IOP lowering, together with robust methods to assess disease progression in terms of functional and structural parameters, although the latter will be challenging in this setting, requiring objective methods such as serial optic disc imaging. The sample size calculation will need to take account of loss to follow up, after pilot testing different approaches to maintain follow up, such as reimbursement of travel expenses, and fast tracking on arrival at the eye clinic.

TDLC controlled IOP in almost three quarters of eyes at 12 months among the 50% of patients who were followed up, with short‐term preservation of vision and minimal complications. Poor follow‐up in this setting highlights the need for an effective, safe and acceptable treatment where regular follow‐up is less critical. Randomized clinical trials of TDLC in Africa are warranted.

## Supporting information


**Table S1**. Transscleral diode laser cyclophotocoagulation treatment studies: indications for and methods of treatment, participants and outcome measures.
**Table S2**. Transscleral diode laser cyclophotocoagulation treatment studies: outcome of treatment and complications.
