# Supplementary material for: Safety and effectiveness of primary transscleral diode laser cyclophotoablation for glaucoma in Nigeria
Source: Clin Exp Ophthalmol. 2018 Jun 17;46(9):1041–7. doi: 10.1111/ceo.13328 (PMC6585748; doi:10.1111/ceo.13328)
Supplement: Supplementary file 1 — Table S1. Transscleral diode laser cyclophotocoagulation treatment studies: indications for and methods of treatment, participants and outcome measures. Table S2. Transscleral diode laser cyclophotocoagulation treatment studies: outcome of treatment and complications. [file CEO-46-1041-s001.docx]

**SUPPLEMENTARY TABLES**

**Table 1:** Transscleral diode laser cyclophotocoagulation treatment studies: indications for and methods of treatment, participants and outcome measures

| **Author, country (ref)** | **Study design** | **Type of glaucoma** | **Indications for treatment** | **Number treated** | **Definition of outcome** | **Preoperative IOP (mmHg) (range)** |
| --- | --- | --- | --- | --- | --- | --- |
| **Studies in Africa** | |  | | | | |
| Egbert,  Ghana(32) | Trial of laser power | POAG | Primary treatment | 92 eyes | Final IOP <22 mm Hg | Mean 29.3 (16-66)mmHg |
| Mavrakanas,  Tanzania(33) | CS: retro-spective | POAG (seeing and non seeing) | Uncontrolled IOP | 49 eyes | Lower IOP | Mean 53mmHg |
| Preussner, Cameroon(34) | CS - prospective using 910nm laser | POAG | IOP reduction; reduction in medication | 272 eyes; 26 followed up | IOP reduction at 1 year | Mean 31.2mmHg |
| Schulze, Malawi(35) | CS: prospective | POAG; PXE | IOP reduction | 47 eyes | Mean IOP reduction | Mean 38.5mmHg |
| **Studies in other countries** | |  | | | | |
| Rotchford, UK(31) | CS: retro-spective | POAG seeing eyes | Primary treatment | 49 eyes | Loss of 2 or more VA line IOP 8-21 | 28mmHg (16-50) |
| Ghosh, UK(30) | CS: prospective | POAG seeing eyes | High IOP | 46 eyes | At 24 months | Mean 24mmHg |
| Kuchar, USA(36) | CS: prospective (micropulse laser) | Advanced | Uncontrolled IOP | 19 eyes | 6-21 mmHg/20% lower at last visit | Mean 37.9 mmHg |
| Butt, Pakistan(37) | Quasi-experimental | POAG | POAG on maximum medication; Primary Rx | 60 eyes | IOP reduction | Mean 41.62 (28 – 60) mm Hg |
| Bloom, UK(38) | CS: prospective. YAG laser | Any type | Refractory glaucoma after multiple procedures | 45 eyes | Not defined | Mean 32mmHg |
| Ansari, UK(39) | CS: retro-spective | Non refractory | Poor control; painful, blind eye allergies; refused surgery; | 74 eyes | Lower IOP fewer medications | Mean 40.3mmHg |
| Spencer, UK(40) | CS: prospective | Refractory | Uncontrolled IOP: surgery refused or unlikely | 58 eyes | Fewer glaucoma mediccations | Mean 33.0 mmHg |
| Martin, UK(41) | CS: prospective | Painful blind eyes | Pain | 30 eyes | IOP reduction; pain relief | Mean 51 mm Hg |
| Kramp, Germany(42) | CS: retro-spective | 109 POAG; 84 secondary | Uncontrolled glaucoma | 193 eyes | IOP 10-22 mmHg | Mean 24.6+/-6.7 mmHg |
| Murphy, UK(43) | CS: retro-spective | Refractory: 46% neovascular | Uncontrolled glaucoma | 263 eyes | IOP <22 mm Hg or > 30% drop in IOP | Mean 40.7 mmHg |
| Lai, Hong Kong(44) | CS: prospective | CACG | Medical uncontrolled CACG | 13 eyes | IOP <21mmHg with or without medication | Mean 36.4 +/- 12.6 mm Hg |
| Grueb, Switzerland(45) | CS: retro-spective | POAG, PXEG | POAG, PXEG | 90 eyes | 4-18mmHg or 20% reduction | Mean 21 mmHg (12–36 mmHg) |
| Vernon, UK(46) | CS: retro-spective | Refractory; 19% 19% | Poor control | 42 eyes | IOP<22; reduction in medicine | Mean 31.4mmHg |
| Iliev, Switzerland(47) | CS: retro-spective | Advanced, refractory: 3% POAG; 61% neovascular | Refractory glaucoma | 131 eyes | IOP 6-21 at last visit | Mean IOP 36.9mmHg |
| Raivio, Finland(48) | CS: retro-spective | 1/3 PXE; POAG/ complex | Poor control; refractory glaucoma | 60 eyes | 8-21mmHg | IOP 27 ± 11 mmHg |
| Frezzoti, Italy(49) | CS: prospective | Advanced/refractory: 36% POAG; 64% complicated | Refractory | 124 eyes | IOP 5-21; pain relief | Mean 29.9 +/- 8.4 mmHg (17-58 mmHg) |
| Zhekov, UK(50) | CS: retro-spective | Refractory. One treatment only.  45 POAG/PACG | IOP maintained; visual acuity | 87 patients | Not defined | IOP 39.5mmHg |

CS = case series; IOP = intraocular pressure; POAG = primary open angle glaucoma; PXEG = primary exfoliative glaucoma; CACG = chronic angle closure glaucoma

**Table 2.** Transscleral diode laser cyclophotocoagulation treatment studies: outcome of treatment and complications

|  | **Follow-up** | **Outcome:**  **Post op IOP** | **Outcome:**  **Visual acuity (change)** | **Complications**  **(n) Number of eyes** | **Comments** |
| --- | --- | --- | --- | --- | --- |
| **Studies in Africa** | | | |  |  |
| Egbert (32) | Mean 13.2 months | ≥20%mmHg drop in 47%; 48% final IOP <22 mm Hg | Worse in 23% but not defined | Atonic pupil (92) 28%; transient hyphaema (3), severe iritis (2). No hypotony, phthisis or sympathetic ophthalmia. |  |
| Mavrakanas (33) | Variable | At last visit 51% eyes had >50% lower IOP | Not reported | No serious complications |  |
| Preussner(34) | 1 year, 26 eyes | Mean reduction: 7.5mmHg | Not reported | No serious complications | Medication reduced from 1.5 to 1.2. |
| Schulze(35) | 3 months | Mean 35.6mmHg | Not reported | Atonic pupil (4) 10.6%; transient iritis (1) (2.1%) | Low dose diode used |
| **Studies in other countries** | | | |  |  |
| Rotchford(31) | 5 years | 79.6% controlled at final follow-up | ≥2 lines: worse 30.6% (15 eyes): 9 glaucoma progression. | Vitreous haemorrhage (1); retinal detachment (1); macula oedema (4) |  |
| Ghosh(30) | 24 months | Mean 17.2 (12-28);  84.8% IOP<21mmHg | >2 lines: same 76.1%; worse 23.9%; (11 eyes); 9 glaucoma progression. | Macula hole (1); retinal detachment (1); macula oedema (2). No hypotony. |  |
| Kuchar(36) | Mean 60.3 days | 22.7 mmHg at last follow-up, 40.1 % decrease. | One line of VA: better 21%; worse 21%. | Hypotony (1). |  |
| Butt(37) | 12 months | Mean 15mmHg at 6 mon; 14.15mmHg at 1 year | Not reported | Anterior uveitis, cataract (8) 13.3% each; hyphaemia (5) 8.3%; hypotony (6) 10% | 45% of eyes retreated. 6% had three treatments. |
| Bloom(38) | No data | Mean 19.3mmHg.  71% treatment success | ≥2 lines: worse 9% | Phthisis (1); chronic hypotony (2); hyphaema and vitreous haemorrhage (1) |  |
| Ansari(39) | 12.5 (4–30) months | Mean: reduced by 45.1% to 21.1 mmHg at final visit. | Mean VA preserved in those with good VA; worse in 13% (glaucoma progression; lens opacity, chronic uveitis) | Hyphaemia (3); chronic iritis (3); corneal oedema (1). No hypotony or other serious complications. | 58% reduction in medication. All with iritis had peripheral iridectomy |
| Spencer(40) | Mean 19 (6-37) months | Mean 16.7 mm Hg at final visit | >2 lines: worse 32% | Rubeotic eye developed endophthalmitis. Chronic hypotony (2) no phthisis (8) 13.3% | Fewer medications: from 2.4 to 1.4. Up to 5 treatment sessions |
| Martin(41) | 6 months | 26 mm Hg. Pain relief in 73.3% | Not reported | Hypotony (3); phthisis (1); uveitis (2); hyphaema (1) |  |
| Kramp(42) | Mean 13.9 (6 to 48) months | Success 76.4%. Mean 19.3+/-5.7 mmHg. Best results in POAG | No reported | Mild anterior uveitis (31); hyphaema (1); phthisis bulbi (3) | ≤6 sessions. 2 phthisical eyes had neovascular glaucoma and PXG |
| Murphy(43) | 17 months (6-46) | Mean 17.7 mmHg. Reduction of 52.6%. Success 89% | Not reported | Hypotony ranged from 0% in POAG to 18.8% in uveitic glaucoma. Persistent uveitis 1.6%: most in complicated glaucoma | A third of eyes were retreated |
| Lai(44) | Mean 26.5 (+/- 4.2) months | Success 92.3%. Mean 18.7 +/- 12.2 mm Hg at final visit | ≥2 lines: same 15.4%; better 46.2%; worse (5 eyes). | No hypotony. Atonic pupil (7) |  |
| Grueb(45) | ≥24 months | Mean 16 (9–27) mmHg. Success 36.7% all  40.9% POAG | Not reported | Hyphaema (1); anterior uveitis (10). No phthisis. |  |
| Vernon(46) | Mean 65.7 (36-84) months | 88.1% success. 50.3% reduction in IOP. Mean 15.6 +/- 6.3 mmHg | Same 26.2%  Better 9.5%  Worse 64.3% | Transient hypotony (2) | Fewer medications: from 2.6 to 1.7. ≤6 sessions. Focus on maintaining acuity |
| Iliev(47) | Mean 30 months (no range) | Success in 69.5%. 45.8% IOP controlled with 1 treatment | Not reported | Hypotony (23) 17.6%. 74% of these eyes had neovascular glaucoma. Chronic uveitis (1); severe dry eye (1); hyphaema (1) vitreous haemorrhage (1). | Fewer medications: mean from 2.97 to 1.39. Multiple eyes were retreated. |
| Raivio(48) | 26 (3–75) months | 18±5mmHg at 6months,  19±7mmHg at 1year,  80% had 30% reduction in IOP at last follow-up | Not reported | Mild anterior uveitis 25%; hyphaema (2 eyes), vitreous haemorrhage (1). No hypotony. |  |
| Frezzoti(49) | 17 (3-42) months | 20.8mmHg (range 6-45) last visit | Loss of two or more lines: 12.9% | Mild anterior uveitis (3) 2.4%; hypaema (2) 1.6%. No hypotony or phthisis |  |
| Zhekov(50) | 3 years | 17.8mmHg at 6 weeks maintained over 3 years | Same or better: 83.6% | Hypotony 5%; no uveitis |  |

IOP = intraocular pressure
